# Cook County Hospital

**Published:** 1877-05

**Authors:** J. H. Wm. Meyer

**Affiliations:** House Physician


### COOK COUNTY HOSPITAL.

(SERVICE OF THOMAS BEVAN, M. D.)

Cases Reported by J. H. WM. MEYER, House Physician.

*Scarlatina and Suppurative Pleurisy.*

Bridget O'C., aged 15, was admitted to the hospital, February 28th, 1877. A week before admission, patient took cold, followed by slight cough. Two days later, she experienced a feeling of general lassitude and weakness, nausea, vomiting and headache. Complained of a dull pain in her right side, and cough.

Four days ago, she became very feverish, pain and cough growing worse, but did not expectorate; the opposite side, also, became painful. At this time, a bright red, punctate eruption made its appearance on her chest.

On admission, patient fairly nourished; tongue coated whitish; bowels move daily; skin very hot, presenting a scarlet rash over the chest, abdomen and thighs; does not eat nor sleep well. Complains of pain in her side and loins. Has been menstruating for four days.

On examination, find diminished movement and dullness over the right chest, posteriorly as high as the spine of the scapula; feeble vesicular respiratory murmur; decreased vocal resonance and fremitus over the same region. Has incontinence of urine during the paroxysms of coughing. Quiniæ sulph., gr. iii. four times daily; spiritus frumenti, tablespoonful every four hours. Also, turpentine stupes to the chest.

March 3d. Bronchial breathing and dullness over the right lung posteriorly to the seventh rib; feeble vesicular murmur below this; broncho-vesicular breathing anteriorly above the fourth rib. Skin desquamating on right side; A. M., temperature, 103; pulse, 134; respiration, 44. P. M., pulse, 144; temperature, 105; respiratory, 40.

March 4th. Did not sleep well; physical signs unchanged. Expectorates a little white, frothy mucus. A. M., pulse, 124; temperature, 102½; respiration, 34. P. M., pulse, 150; temperature, 105; respiration, 42.

March 5th. Bronchial breathing heard posteriorly over right lung, from apex to lower angle of scapula; anteriorly broncho-vesicular breathing, with some large and small mucus rales. Breathing very difficult. A. M., pulse, 150; temperature, 102½; respiration, 52. P. M., pulse, 140; temperature, 102½; respiration, 40.

March 6th. Was delirious all night. A. M., pulse, 138; temperature, 98½; respiration, 36. P. M., pulse, 138; temperature, 102½; respiration, 40.

March 7th. Expectorates large quantities of tenacious yellow sputum. A. M., pulse, 140; temperature, 98; respiration, 44. P. M., pulse, 136; temperature, 100; respiration, 40.

March 8th. Find moist rales all over left lung. Urine still escapes during the paroxysms of coughing. A. M., pulse, 130; temperature, 103$\frac{3}{4}$; respiration, 39. P. M., pulse, 150; temperature, 102$\frac{1}{2}$; respiration, 44.

March 9th. Expectorates excessively. Examination of sputum with microscope show it to be composed almost entirely of pus. Find amphoric resonance, breathing and voice over left scapular and interscapular region, A. M., pulse, 136; temperature, 99; respiration, 34. P. M., pulse, 134; temperature, 103$\frac{1}{2}$; respiration, 34. Some of the sputum was boiled with liq. potassæ; but no elastic tissue could be found.

March 11th. Died.

An examination was made ten hours after death. The right lung was much shortened, leaving a space bounded above by the lower lobe, below by the pleura, covering the diaphragm externally by a false membrane, which was firmly adhesive to the parietal layer of the pleura, within which space was contained at least a quart of purulent fluid. This fluid extended up as far as the pleural reflection at the root of the lung.

In the lower lobe of the right lung was a small cavity, which communicated externally with the pleural cavity, internally with a bronchiole. Both cavity and bronchiole contained purulent fluid. Over the lower lobe of the left lung, some adhesions to the diaphragm were found, with some fluid in the pleural sac. Parenchyma of right lung was compressed, and gave diminished crepitation, yet floated upon water. On section, found increased amount of mucus, with a little pus. The condition of the left lung was similar, except that there was less compression and no pus.

---

*Phthisis and Empyema.*

Claus Cl., native of Denmark, cabinet-maker, aged 33 years, was admitted, February 7th, 1877. There is no record of consumption in patient's family history. Has himself enjoyed good health until last May; at that time he took cold after drinking cold water on a very hot day. He contracted a cough, from which he has never had entire relief; had pains in his chest. During the summer his health gradually failed, appetite being variable; bowels not regular; had scanty expectoration of white frothy mucus. During September he began to suffer from occasional night sweats, a tickling sensation in the throat, huskiness of voice, dyspnœa; sputum became yellowish in color. One night he awoke to find blood running from his mouth; thinks he lost half a pint at that time. Was in this hospital during last November, during which time his symptoms were much improved. On leaving, however, he took a fresh cold, and suffered a relapse to his former condition. On admission is somewhat emaciated; skin normal; face flushed; tongue large, coated white; appetite poor; bowels regular; urine of high color, and offensive odor; pulse, 120; respiration, 24; decubitus on left side; coughs constantly, expectorates a greenish yellow sputum; has dyspnœa and night sweats. On physical exploration, find loss of vocal fremitus over lower half of right chest.

On percussion, patient in recumbent position, find slight dullness just below right clavicle; then fair resonance as far as fifth rib, in front; dullness in axillary region, and posteriorly; patient lying on his breast, find resonance posteriorly; dullness in front almost to clavicle, fixed dullness anteriorly over left lung as far as fifth rib; fair resonance posteriorly. On auscultation, find low-pitched, blowing respiration, just below right clavicle; broncho-vesicular, below that; exaggerated high-pitched respiration on left side anteriorly. Behind find feeble respiratory murmur over lower half of right lung; exaggerated respiration with some broncho-vesicular breathing over left. Withdrew and examined a few drops of fluid from chest; find pus-corpuscles in abundance.

March 12th. Feet are slightly œdematous.

March 18th. Is expectorating purulent liquid at the rate of about an ounce per hour.

March 19th. Slept well; feels pain in right side; upon coughing notices a particularly offensive odor of the breath.

March 20th. Still expectorating purulent liquid profusely.

March 21st. Get well marked, amphoric breathing and voice, and succussion.

March 27th. Gradually failing. Still expectorates profusely.

March 29th. Died.

Post mortem ten hours after death. Found about a gallon of pus in right pleural sac. The pleural surfaces were very thickly covered by false membrane. The apex of right lung was firmly adherent to the chest; the other parts floated on the pus. The upper lobe of left lung was infiltrated by a deposit; the right apex contained several small cavities, one of which communicated with the pleural sac. All the other organs were normal.

---

*Cancer of the Lung.*

Charles L., native of Sweden, aged 30, a carpenter, was admitted February 10th, 1877.

In June, 1875, patient's left leg was amputated at the lower third, for cancer of the foot, in St. Joseph's Hospital.

Five weeks previous to admission he took cold; had a slight cough, but was not confined to his bed until three weeks later, when he complained of dizziness; pain in the left side of the chest; and cough; expectorating large quantities of white frothy sputum. A week ago he began to expectorate small quantities of blood.

On admission, poorly nourished; tongue coated whitish. The skin presents a peculiar sallow hue. Find a small tumor on the left leg, midway between the stump and knee; the mammae enlarged, hard, and indurated. The left side of the chest measures one half inch more than the right. Apex beat of the heart in the epigastric region. Examination of the chest reveals dullness on the left side as high as the inferior angle of the scapula; decreased vocal fremitus and respiratory murmur; also friction sound in the left inflammatory region.

February 20th. Dullness extends somewhat higher. Gradual enlargement of the tumor on the leg.

March 6. Expectorated about three ounces of bright red frothy blood this morning.

March 19. Has great dyspnœa; is compelled to sit up in bed; coughs constantly, expectorating white frothy sputum, occasionally streaked with blood. Tumor on the leg growing rapidly.

March 25. Amphoric respiration over the apex of the left lung.

March 28th. Died.

Autopsy twelve hours after death. Slight adhesions were found between pulmonary and costal pleuræ over the entire left lung. A few also on the right side.

The anterior border of the left lung extended about one and one-half inches to the right of the median line. The heart was displaced considerably to the right. As the root of the lung was severed, exit was given to a large quantity of brain-like matter, mixed with blood. The lung was almost twice as large as the normal organ. Disseminated through both lungs were masses of brain-like matter, varying from the size of a pea to that of an orange. These were especially numerous and large in the left lung. The mesenteric glands were enlarged, and mesenteric vessels congested. The rectum and greater part of descending color were considerably constricted in size. The tumor on the leg, involving the bone to some extent, was composed of this same brain-like matter.

---

*Atheromatous Degeneration.*

William A., American, carpenter, aged 64 years, was admitted to the hospital, March 7th, 1877.

As the patient was somewhat delirious, we could get no history, but learned from friends that four weeks previous to admission he was suddenly seized with dizziness; vertigo lasting about an hour; since then he has been lethargic, and at times delirious. On admission, well-nourished tongue moist and clean; appetite and sleep fair, bowels regular; surface normal; pulse feeble, 90 per minute. The radial artery is tortuous and moves under the finger as a hard cord.

March 18th. Hands and feet are very cold, head hot. Passes urine and fæces in bed; is still delirious.

March 22d. Patient gradually failed and died to-day.

An examination was made ten hours after death. In removing the calvarium, about three ounces of a yellowish white fluid escaped. The dura mater was greatly thickened and firmly adherent to the extent of about one inch on either side the median line of the calvarium. The pia mater was thickened and congested. The arteries at the base of the brain were in some portions sacculated, in others contracted, and contained numerous clots. Their walls were thickened, and at many points were found deposits of a hard whitish substance. The ventricles were enlarged and contained considerable fluid.

The arch of the aorta was greatly roughened by calcareous deposits, being most marked in the descending portion; in places small abscesses were found. The valves of the heart were normal. Coats of all the arteries examined in various parts of the body seemed to be thickened. The costal and pulmonary pleuræ of the right lung quite extensively adherent, and the lung in a state of senile pneumonia.

